# Apple Fruit Growth and Quality Depend on the Position in Tree Canopy

**DOI:** 10.3390/plants11020196

**Published:** 2022-01-12

**Authors:** Darius Kviklys, Jonas Viškelis, Mindaugas Liaudanskas, Valdimaras Janulis, Kristina Laužikė, Giedrė Samuolienė, Nobertas Uselis, Juozas Lanauskas

**Affiliations:** 1 Institute of Horticulture, Lithuanian Research Centre for Agriculture and Forestry, Kauno Str. 30, LT-54333 Kaunas, Lithuania; jonas.viskelis@lammc.lt (J.V.); kristina.lauzike@lammc.lt (K.L.); sdi@lammc.lt (G.S.); nobertas.uselis@lammc.lt (N.U.); juozas.lanauskas@lammc.lt (J.L.); 2 Department of Horticulture, Norwegian Institute of Bioeconomy Research—NIBIO Ullensvang, Ullensvangvegen 1005, NO-5781 Lofthus, Norway; 3 Laboratory of Biopharmaceutical Research, Department of Pharmacognosy, Lithuanian University of Health Sciences, Sukilėlių Av. 13, LT-50162 Kaunas, Lithuania; mindaugas.liaudanskas@lsmuni.lt (M.L.); farmakog@lsmuni.lt (V.J.); 4 Institute of Pharmaceutical Technologies, Lithuanian University of Health Sciences, Sukilėlių Av. 13, LT-50162 Kaunas, Lithuania

**Keywords:** bioactive compounds, irradiance, *Malus* × *domestica* Borkh., maturity indices, photosynthetic indices, radical scavenging activity

## Abstract

Modern apple orchard systems should guarantee homogeneity of fruit internal and external qualities and fruit maturity parameters. However, when orchards reach productive age, a variation of these parameters takes place and mostly it is related to uneven light distribution within the tree canopy. The aim of the study was to evaluate the canopy position’s effect on fruit internal and external quality parameters. This is the first study where all the main fruit quality and maturation parameters were evaluated on the same trees and were related to the light conditions and photosynthetic parameters. Four fruit positions were tested: top of the apple tree, lower inside part of the canopy, and east and west sides of the apple tree. Fruit quality variability was significant for fruit size, blush, colour indices, total sugar content, dry matter concentration, accumulation of secondary metabolites and radical scavenging activity. Fruit position in the canopy did not affect flesh firmness and fruit maturity parameters such as the starch index, Streif index and respiration rate. At the Lithuanian geographical location (55°60′ N), significantly, the highest fruit quality was achieved at the top of the apple tree. The tendency was established that apple fruits from the west side of the canopy have better fruit quality than from the east side and it could be related to better light conditions at the west side of the tree. Inside the canopy, fruits were distinguished only by the higher accumulation of triterpenic compounds and higher content of malic acid. Light is a main factor of fruit quality variation, thus all orchard management practices, including narrow two-dimensional tree canopies and reflecting ground covers which improve light penetration through the tree canopy, should be applied.

## 1. Introduction

Facing overproduction of apples in world markets, fruit quality is becoming a main issue to enlarge the consumption of apples and thus increase benefits to growers. Many technological, biological and environmental factors affect apple fruit quality. Our previous studies demonstrated the significant impact of rootstock [1], planting distances [2], tree crown management and tree growth control [3,4], fertilization [5], crop load management [6], orchard location [7], etc., on external and internal fruit quality parameters. Most of these practices could be attributed to the improvement of light conditions and to the increase of light interception in modern orchards.

Light is the essential factor to achieve proper fruit quality and high yields [8]. Robinson et al. [9], aiming on the highest orchard productivity, suggested that light interception should reach 70–75%. Similar levels were reported by other researchers in order to maintain high and annual apple yields [10]. Higher light interception could be achieved by high density planting systems [11]. 

Modern apple orchard systems should guarantee not only high yields but also homogeneity of fruit internal and external qualities and fruit maturity parameters. However, when orchards reach productive age, a variation of these parameters takes place and mostly it is related to uneven light distribution within a single tree canopy. Apples at various canopy positions are exposed to different irradiance levels. These differences create different environments for fruit development and these lead to varying fruit appearances and varying internal qualities. Transportation of mineral nutrients depends on the leaf and fruit transpiration rate, and it was found that sun-exposed fruits with a higher transpiration rate accumulate more minerals than fruits in shade conditions [12,13,14]. The fruit position in the tree can affect accumulation of the primary and secondary metabolites in the fruit. Leaves in the outer canopy receive and intercept much more light resulting in the enhanced synthesis of photoassimilates [15], which convert into a higher carbohydrate accumulation in fruits [16]. Therefore, fruits from the outer canopy contain higher concentrations of sugars, soluble solids and dry matter [17]. Low light conditions inside the canopy can result in worse colouration of apples [18], but on another hand, too high irradiance can cause physiological disorders such as sunburn damage or fruit rotting [19,20]. 

Most of the mentioned studies present results on the separate fruit quality parameters. There are no investigations performed where all main fruit quality and maturation parameters are evaluated on the same trees and are related to the light conditions and photosynthetic parameters.

Apple tree growth and yield strongly depend on the latitude [21]. The solar zenith at 55° North altitude is at a much lower angle than in the main apple growing regions. Changes of light quality, day length and irradiance level are decisive factors to achieve high fruit quality parameters. Such types of studies in high latitudes have not been performed. In our study, we investigated the intra-canopy variability of fruit development. The aim of this study was to evaluate the effect of fruit growth position in the apple tree canopy on growth, maturation and the internal and external fruit quality parameters.

## 2. Results and Discussion

### 2.1. Light Conditions

Absolute PAR values varied between 700 and up to 2200 μmol m^−2^ s^−1^ depending on the time of the year and on the time of the day when the light records were taken. Weather conditions (sunny vs. cloudy) during the measurements had an even bigger influence on PAR data. In order to discard the weather factor, every measurement was conducted simultaneously taking records at canopy positions and recording open sky value. Comparing open sky value and available light at different canopy positions and expressing this difference in percentage allowed us to make comparisons between various canopy positions, disregarding weather conditions during the records. 

**Figure 1:**
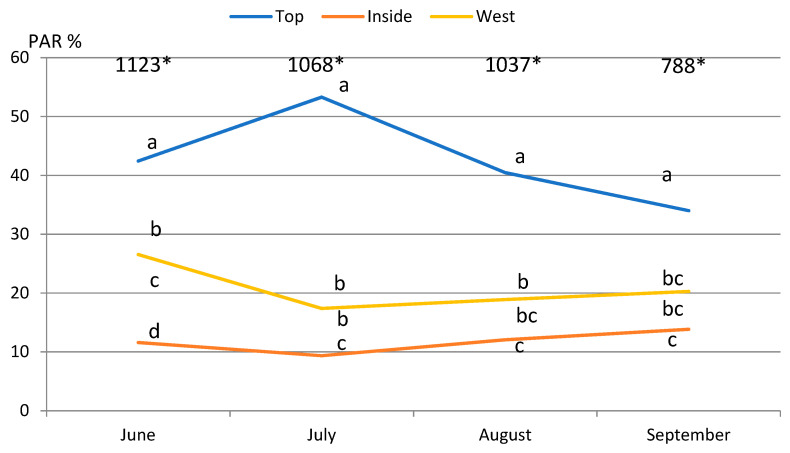
Available photosynthetically active radiation in different positions of the canopy, % from full light, average 2018–2020. The different letters at the same date indicate significant differences between canopy positions (Tukey’s (HSD) multiple range test at the confidence level *p* = 0.05), * average of open sky value, μmol m^−2^ s^−1^.

Available light varied highly in the tree canopy. During all the measurement dates the highest percentage, significantly, of photosynthetically active radiation (PAR) was recorded at the top of the canopy where it varied from 53% in July to 34% in October (Figure 1). On average during the growing season, the top of the canopy receives 43% of the available light, while inside of the canopy receives only 12%. Our data are similar to what was reported by other trials. Tustin et al. [22] indicated that the inside of the canopy receives 13%, when the outer canopy receives 54% of the open sky value. Fouche et al. [23] indicated that only 2% of available light reaches the inside of the tree canopy. We noticed that irradiation of the west and east side of the tree canopy was different at the Lithuanian geographical location. On average during the growing season, the west side receives 21% and the east side 16% of the available light. The highest irradiance was recorded in June, except the top of the tree, when the foliage was still not fully developed and new shoots had just started to grow. Starting from July, light conditions in all canopy positions stabilized and remained the same until the end of the growing season. The decrease of available light was recorded only in the top of the tree where there was the strongest shoot growth.

### 2.2. Photosynthetic Parameters

The canopy position had a significant effect on photosynthetic parameters. Light absorbance by leaves and penetration through the canopy is one of the most important factors affecting photosynthesis and metabolism [24,25]. Photosynthetic indices tend to decrease with decreasing light intensity due to the foliage and sun position. Leaf stomatal conductance was up to 67% higher, the photosynthetic rate up to 70% higher and transpiration up to 37% higher on the top of the canopy compared to the inside of the canopy (Table 1). 

The chlorophyll index (Chl) and the nitrogen balance index (NBI) measure the relative amount of chlorophyll and nitrogen at the same point on the leaf in the same time moment and may convey N dynamics in apple tree systems [26,27]. The NBI status of leaves has a relationship to light availability and light limitation [28]. Significantly, the highest index of chlorophylls and flavonoids was found in the top of the canopy. The photosynthetic indices in the top of the canopy, which received the strongest and longest luminous flux during the day, were significantly higher and the obtained data fully agree with other researchers indicating that these indices are directly dependent on the level of illumination [25].

The photosynthetic rate was significantly higher on the east side compared to the west side. Previous studies show that the highest photosynthetic activity is in the first half of the day [29], so the leaves on the east side are adapted to more active photosynthetic activity than the leaves on the west side, which receive more sunlight when the photosynthetic activity of the plant decreases.

According to the obtained results, the nitrogen balance index (NBI) was significantly higher in the lower part of the canopy compared to the fully lightened leaves on the top and on the east part of the canopy. These data correspond to the findings by Cronin and Lodge [30], as they found that low light availability increased the nitrogen content of leaf tissue by 53%. Canopy position has no effect on intercellular CO_2_.

### 2.3. Fruit Share by Canopy Positions

During the study, the average yield per tree was 28.9 kg. Fruit yield was relatively evenly distributed in different canopy positions, and a significant difference was found only between the top and inside of the canopy (Table 2). The study was conducted when the trees were fully developed and reached the projected space in the orchard. Shading inside the canopy could inhibit flower bud formation which led to lower yields.

### 2.4. Fruit Weight and Diameter

Apples from the top had significantly higher fruit weight compared with other canopy positions, which is in line with other studies [31]. Average top fruit weight exceeded the weight of inside fruits by 24%, and by 17% the weight of fruits from west and east canopy positions (Table 2). Even greater differences were recorded for fruit diameter. The share of fruits of first class (diameter >65 mm) in the top position reached almost 96%, when the share of fruits inside the canopy was only 63%. There were no differences in fruit weight and diameter between fruits from the west and east canopy positions, but both positions differed significantly from the inside position. According to Corelli-Grappadelli and Lakso [32], fruit growth rates are correlated to the temperature regimes. Due to higher irradiance, there are higher temperatures in the tree top and in the outer canopy that enhance faster fruit growth in the early season and result in larger fruit size and weight during the harvest.

### 2.5. Fruit Blush and Colour

Significant differences among positions were recorded evaluating fruit blush. Red blush covered 67% of the top fruit surface and only 22% of the inside fruit surface (Table 2). Worse colouring of bi-coloured cultivar results in a lower fruit quality class and lower consumer preference [33], as well as lower market price and economic losses to producers. It is well known that apple colour development could be increased by better fruit exposure to the light [18]. Comparing fruits from the west and east positions there were no significant difference in fruit blush cover, but a tendency of better colouring of fruits from the west position was noticed in some years of the study. The reason could be a better illumination of the west side of the tree.

Colour space coordinates (CIE L*a*b* system) revealed differences of colour pattern and intensity between fruits from various canopy positions (Table 3). Significant differences were established between the colouring of inside fruits and fruits from other positions. The green–red component a* and yellow–blue component b* indicated that inside fruits were more greenish and yellowish. Hue angle h° and lightness value L* indicated that top fruits significantly had the most intense and dark red colour compared to all other canopy positions. Colour space coordinates did not disclose any differences in colour pattern between fruits from the east and west canopy positions. 

### 2.6. Fruit Maturation

Despite the differences in external fruit appearance, canopy position did not affect fruit maturity indicators such as starch index, Streif index, fruit respiration rate and flesh firmness (Table 2). Similar results were provided by the study conducted in Italy [34]. 

Only one of the fruit maturity parameters—index of absorbance difference (*I_AD_*)—differed significantly between inside canopy fruits and fruits from other canopy positions. Our results confirmed findings of Cocetta et al. [35] that *I_AD_* values of fruits exposed to the sun are higher than those of the shaded side.

### 2.7. Fruit Biochemical Content

Leaves and fruit in the outer canopy receive and intercept much more light resulting in the enhanced synthesis of photoassimilates [15], which converts into higher carbohydrate accumulation [16]. Palmer et al. [36] proposed fruit dry matter (DM) content as a new quality parameter for apple fruits, providing a positive relationship between DM and consumer preference. DM content is related to the total amount of sunlight intercepted [11], so top fruits and fruits from the west side of the row had significantly higher DM than inside fruits (Table 4), which was stated in other studies [37].

In our study, fruit soluble solid content (SSC) did not differ significantly among canopy positions (Table 4). These findings contradict some studies where it was stated that shading reduced SSC in peaches [38], cherries [39] and apples [37,40]. On the other hand, top fruits had the highest total sugar content. Endrizzi et al. [41] stated that overall liking of apple fruits was positively influenced by high levels of sweetness. Significant differences were found comparing top and inside fruits, but no differences were found comparing total sugar content of these fruits with fruits from the west and east sides of the tree canopy.

Fructose, sucrose and glucose are the major soluble sugars in apples, and sorbitol is a main sugar alcohol [42]. Higher absorbance of PAR by the leaves resulted in enhanced synthesis of photoassimilates in the outer canopy. Sorbitol and sucrose are the main photosynthetic products and translocation sugars in apples [43]. Though there were no significant differences in the average sucrose content between fruits from the top, west and east positions, significantly lower sucrose accumulation in inside fruits was established (Table 5). The accumulation of sorbitol was even more dependent on irradiance level. Sorbitol content in fruits from the top and west side of the canopy was significantly higher than in fruits from the east side. The lowest sorbitol content was found in inside fruits. Canopy position did not have an effect on fruit fructose content, but the highest glucose content was found in fruits from inside the canopy. In addition, inside fruits accumulated, significantly, the highest content of malic acid too. Malic acid is primarily responsible for fruit acidity in apples [44]. The higher acidity of fruits from the shaded part of the canopy was also established by Hamadziripi et al. [33], Nilsson and Gustavsson [37] and Robinson et al. [16], though there are studies stating that canopy position has no effect on malic acid concentrations [45].

Anthocyanins are mainly responsible for fruit colour development and their synthesis is directly related to light conditions [46]. Higher light conditions and cool temperatures promote anthocyanin synthesis in fruit peels [23,47]. These findings are based on recent molecular studies [48,49]. In addition, it was established that the MdMYB1 gene plays a critical role in regulating anthocyanin synthesis in apple fruits. Honda and Moriya [50] developed a marker-assisted selection process to identify MdMYB1 genotypes and predict those fruits that will develop redder skin. Sun et al. [51] found that R2R3-type MYB transcription factor *MdMYB90-like* is responsible for improved apple colouring. Recent molecular studies and new apple breeding methods led to the creation of apple cultivars with enhanced colouring in various light and temperature regimes.

Most of the studies indicate that apple peel from the sun-exposed outer canopy is higher in anthocyanin concentrations than inner canopy fruit [52,53]. In our study, the total anthocyanin content in the peel of fruits from the tree top exceeded the content of fruits from the inside of the canopy even by nine times (Table 6). These fruits accumulated 2–3 times less anthocyanins that fruits from the east and west sides of the tree. Fruits from the west side of the canopy which receive higher irradiance throughout the growing season accumulated more anthocyanins than fruits from the east side. The main anthocyanin pigment responsible for the fruit skin’s red colour is cyanidin-3-galactoside. Canopy position determined its content in the same way as the total anthocyanin content (Table 7). Cyanidin content did not differ significantly in fruit peel from inside, east and west positions but top fruits accumulated a significantly higher amount. Meanwhile, other anthocyanins such as cyanidin -3-glucoside and cyanidin -3-arabinoside were not even detected in fruits from inside and the east side of the canopy.

Research on the effects of location within the canopy on both primary and secondary metabolites showed the importance of light exposure on apple fruit quality [37,45,54,55]. The highest total phenol content was found in top fruits but it differed significantly only from inside fruits and fruits from the west side of the canopy (Table 6). There were no significant differences in total phenol content comparing inside, west and east canopy positions. Investigating the composition of phenolic compounds, top fruits accumulated significantly higher amounts of the majority of quercetin glycosides (hyperoside, isoquercitrin, quercitrin and rutin) and procyanidin B2 than fruits from other canopy positions (Table 8). Canopy position did not affect the content of chlorogenic acid and (−)-epicatechin.

Apples, from the inside of the canopy, accumulated the highest amount of total triterpene compounds and differed significantly from fruits from the top and west side of the canopy (Table 6). Our data show that the concentration of triterpene compounds could be attributed to solar irradiation and is negatively related to the amount of received sunlight. The data from other studies indicate that differences of irradiance levels between sun-exposed and shaded fruits cause a different fruit wax formation and its structure [56,57], and that could explain the higher content of triterpene compounds in fruit peel from the inside and east side positions, where illumination is lower. Investigating the composition of triterpene compounds, significant differences were found in betulinic, ursolic and oleonolic acid content (Table 9). Only the content of corosolic acid was less dependent on fruit canopy position.

Higher amounts of bioactive compounds in fruits from the top and west canopy position determined higher radical scavenging activity (Table 6). These findings are in the line with the studies of Drogoudi and Pantelidis [54] and Hamadziripi et al. [33] where the antioxidant contents were greater in the upper positions of the tree canopy.

Most of the current studies are orientated to the increase of light interception and orchard productivity. Reduced spacing within and between the rows and increased tree heights increase orchard productivity, but can lead to increased shading and not even distribution of light across the tree canopy. The proper illumination of a canopy is a function of the canopy size (interception) and shape (distribution) [58]. Our study revealed that high density plantings and three-dimensional canopy shape do not guarantee the same growth conditions at various canopy positions and increase fruit quality variation. Due to tree shading, twenty percent of fruits are lacking external quality and have a lower nutritional and pharmaceutical value.

**Figure 2:**
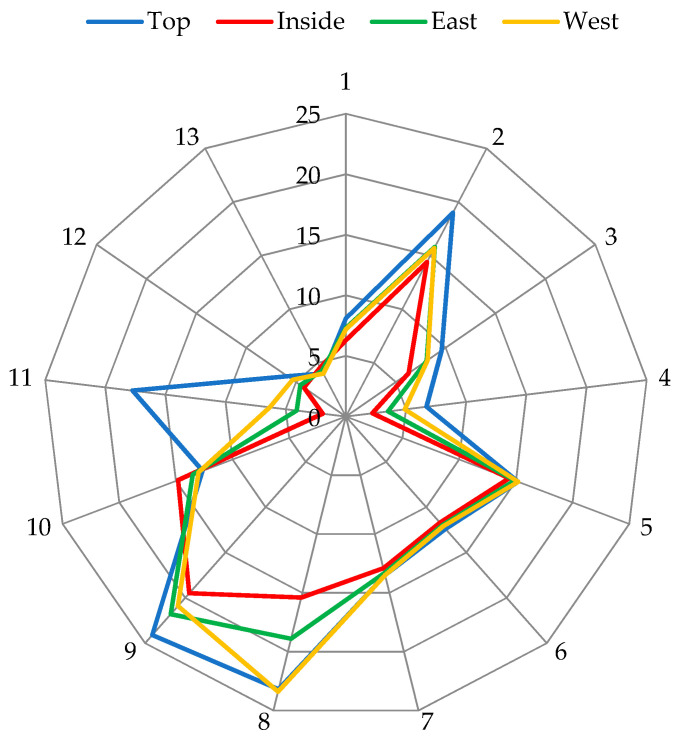
Effect of canopy position on the internal and external quality parameters of apple fruit, where 1—fruit yield, kg; 2—fruit weight, g (divided by 10 (/10)); 3—share of fruits with diameter >65 mm, % (/10); 4—fruit blush, % (/10); 5—dry matter content, %; 6—total sugar content, %; 7—soluble solid content, %; 8—sorbitol content, mg g^−1^ DW; 9—total phenolic content, mg g^−1^ DW (multiplied by 10); 10—total triterpene content, mg g^−1^ DW; 11—total anthocyanin content µg g^−1^ DW (/10); 12—radical scavenging activity (TE_ABTS_, μmol/g); 13—malic acid content, mg g^−1^ DW (/10).

In high altitudes, due to light conditions during the growing season there is a great variation of the main fruit quality parameters even in the modern orchards. Different irradiance levels cause differences in photosynthetic parameters and lead to varying fruit development and uneven accumulation of bioactive compounds. Almost all tested fruit quality parameters of fruits from the top of the apple tree canopy exceeded the quality of fruits from other positions (Figure 2). Comparing fruit quality from the west and east side of the canopy, a tendency of higher fruit quality from the west side was noticed. Canopy position did not affect fruit ripening parameters.

Inside canopy leaves were distinguished by a significantly lower photosynthetic rate, stomatal conductance and transpiration rate compared to other canopy positions. Therefore, fruits which grow inside of the tree canopy and make up to 20% of the total yield have significantly lower fruit quality: smaller size and weight, worse colouring, lower content of anthocyanins, sorbitol and sucrose and lower radical scavenging activity. Shade conditions favoured only the accumulation of triterpenes and higher concentration of malic acid (Figure 2).

Our study revealed that a light is the main factor of fruit quality variation, thus all orchard management practices, including two-dimensional tree canopies [59], and reflecting ground covers [60,61], which improve light penetration through the tree canopy should be applied.

## 3. Materials and Methods

### 3.1. Experimental Design

The study was carried out at the Institute of Horticulture, Lithuanian Research Centre for Agriculture and Forestry (55°60′ N, 23°48′ E) during 2018–2020. Apple cultivar ‘Ligol’ on rootstock P 60 planted at a distance of 4 × 1.25 m in 2002 was studied. Orchard row direction was south-north. Trees were trained as slender spindles. A sustainable plant protection system was used for orchard management [62].

**Figure 3:**
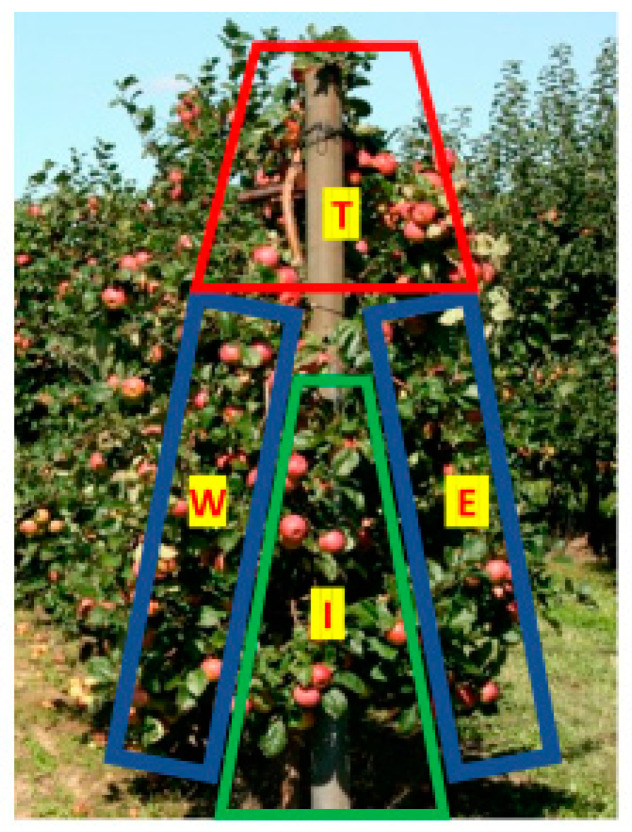
Ilustration of tested canopy positions, where T—top part of the canopy, W—west side, E—east side, I—inner part of the canopy (inside).

Four fruit positions in the canopy were tested: (1) top of the apple tree (T) (above 1.8 m), (2) lower inside part of the canopy (I) and (3–4) east (E) and west (W) sides of apple trees (Figure 3). Apples for the last two treatments were harvested at 1.2–1.8 m above the ground. The trial was established in six replicates with two apple trees per plot. The experiment was laid out in a completely randomized design. Ten fruits per each apple tree from the tested fruit position in the canopy were randomly selected for the analysis. Totally, 20 fruits per replicate were analyzed at optimum harvest time.

### 3.2. Light Measurements

Light conditions in different canopy positions were measured in monthly intervals 3 times per day in the morning (8 a.m.), noon (12 a.m.) and afternoon (4 p.m.). Recording was started from the begining of June when fruitlets reached 15–20 mm size and ended at fruit harvest time in October. Photosynthetically active radiation (PAR) was measured by an AccuPar LP80 device (Decadon devices, Pullman, WA, USA) with 86 cm length probe equiped with 80 light sensors and simultaneously with an external above canopy sensor. Available PAR at different canopy positions was expressed in percents of full light. PAR of the top of the canopy was evaluated at 1.8 m holding the middle of the probe at the tree trunk, west and east sides of the canopy, at 1.2 m holding the end of the probe at the tree trunk, and inside canopy measurements were recorded at 1 m holding the middle of the probe at the tree trunk. The mean of three records at each canopy position was counted as the replicate.

### 3.3. Fruit Colour

Colour indexes in the space of even contrast colours were measured with a spectrophotometer MiniScan XE Plus (Hunter Associates Laboratory, Inc., Reston, VA, USA), and chroma (C = (a*^2^ + b*^2^)^1/2^) and hue angle (h° = arctan(b*/a*)) were calculated.

The fruit cover colour (blush) was evaluated in a 1–9-point scale, where 1—0% of blush, 9—100% of fruit surface was covered by red blush.

### 3.4. Fruit Chemical Content

Soluble solids were quantified with a digital refractometer PR-32 (Atago Co., Ltd., Tokyo, Japan).

Fruit firmness was determined by the TA.XTPlus texture analyzer (Stable Micro Systems, Godalming, UK) using the P/2 probe.

Dry matter content was determined gravimetrically by drying apple samples to a constant weight at 105 °C.

### 3.5. Fruit Maturity Indexes

The starch index was determined using a 4% potassium iodide and 1% iodine solution (scale 1–10).

The Streif index was calculated as F/RS (F—firmness, R—soluble solids concentration, S—starch conversion).

The handheld delta absorbance (DA) meter (Sinteleia, Bologna, Italy) was used to measure chlorophyll content in fruits and provide an index of absorbance difference (*I_AD_)*. *I_AD_* was measured at harvest on two opposite fruit sides.

The fruit respiration rate was measured with a gas analyzer EASI-1 (Absoger, Les Barthes, France).

### 3.6. Photosynthetic Parameters

The nitrogen balance index (NBI), chlorophyll index (Chl) and flavonols index (Flav) were evaluated using a no-destructive measurement of leaf chlorophyll and flavonoid content (related to nitrogen/carbon allocation) in the epidermis (Dualex ^®^4, Dynamax Inc., Houston, TX, USA).

Photosynthetic parameters (photosynthetic rate, stomatal conductance, transpiration and intercellular CO_2_) were determined at 9:00–12:00 a.m. and 13:00–14:00 p.m. by using an LI-6400XT portable open flow gas exchange system (Li-COR Biosciences, Lincoln, NE, USA). Reference air [CO_2_] (400 μmol mol^−^^1^), light intensity (1000 μmol m^−^^2^ s^−^^1^) and the flow rate of gas pump (500 mmol s^−1^) were set. Measured for 1 min every 3 s on each leaf, the measurement began with the complete stabilization of the indicators. Measurements were made on the same leaf with both systems.

### 3.7. Preparation of Apple Extracts for the Determination of Phenolic Compounds

Apples were cut into slices and stalks and the seeds were removed. The apple slices were immediately frozen and later lyophilized in a Zirbus lyophilizer (Zirbus Technology GmbH, Bad Grund, Germany) at 0.01 mbar pressure and with −85 °C condenser temperature. The amount of 2.5 g of lyophilized apple powder (exact weight) was weighed, added to 30 mL of ethanol (70%, *v/v*) and extracted in a Sonorex Digital 10 P ultrasonic bath (Bandelin Electronic GmbH, Berlin, Germany) for 27 min at 45 °C. The extract obtained was filtered through a paper filter; the apple lyophilizate on the filter was washed twice with 10 mL of ethanol (70%, *v/v*) in a 50 mL flask. The extract was filtered through a membrane filter with a pore size of 0.22 µm (Carl Roth GmbH, Karlsruhe, Germany).

### 3.8. Preparation of Apple Extracts for the Determination of Triterpenic Compounds

Apples were peeled and the peel was immediately frozen and later lyophilized. The amount of 1 g of lyophilized apple peel powder (exact weight) was weighed, added to 10 mL of acetone (100%, *v/v*) and extracted in a Sonorex Digital 10 P ultrasonic bath (Bandelin Electronic GmbH, Berlin, Germany) for 10 min. The conditions of extraction (type of extraction, duration, temperature, solvent and its concentration) were chosen considering the results of extraction optimization. The extract was filtered through a membrane filter with a pore size of 0.22 µm (Carl Roth GmbH, Karlsruhe, Germany).

### 3.9. Preparation of Apple Extracts for the Determination of Anthocyanins

Fruits were collected and subjected to freeze-drying. The dried fruits were milled to powder and kept in a sealed container in a dark dry place. The amount of 2.5 g (exact weight) of lyophilized apple powder was weighted in a dark glass vial, added to 25 mL of 70% (*v/v*) ethanol acidified with 2% hydrochloric acid. The extraction process continued for 20 min at 80 kHz and 1130 W, in an ultrasonic bath (Elmasonic P, Singen, Germany). The extracts were filtered through 0.22 μm pore size membrane filters (Carl Roth GmbH, Karlsruhe, Germany).

### 3.10. Preparation of Apple Extracts for the Determination of Sugars and Malic Acid

An amount of 0.5 g of lyophilized apple powder (exact weight) was weighed, added to 50 mL of purified water. Extraction was performed for 10 min while shaking (VWR^®^ Mini Shake, VWR International, Radnor, PA, USA). The extract was filtered through a membrane filter with a pore size of 0.22 µm (Carl Roth GmbH, Karlsruhe, Germany).

### 3.11. High Performance Liquid Chromatography (HPLC) for the Determination of Phenolic and Triterpenic Compounds

Phenolic compounds (hyperoside, isoquercitrin, rutin, reynoutrin, avicularin, quercitrin, procyanidin B2, procyanidin C1, (+)-catechin, (−)-epicatechin, phlorizin and chlorogenic acid) were determined through the high-performance liquid chromatography (HPLC) method, as described in the article by Liaudanskas et al. [63]. Triterpenic compounds (corosolic, betulinic, oleonolic and ursolic acids) were determined through the HPLC method, as described in the article by Butkevičiūtė et al. [64].

### 3.12. Ultra-High Performance Liquid Chromatography (UHPLC) for the Determination of Anthocyanin Compounds

The variability in the qualitative and quantitative content of anthocyanins was evaluated by the validated method [65]. Chromatographic separation was performed with Waters ACQUITY Ultra-High Performance LC system (Water, Milford, MA, USA) equipped with a photodiode array detector and an ACE Super C18 (100 × 2.1 mm, 1.7 μm) column (ACT, Aberdeen, UK). The gradient elution system consisted of 10% (*v/v*) formic acid in water (A) and 100% (*v/v*) acetonitrile (B), and separation was achieved using the following gradient: 0–2 min, 5–9% B; 2–7 min, 9–12% B; 7–9 min, 12–25% B; 9–10 min, 25–80% B; 10–10.5 min, 80% B; 10.5–11 min, 80–5% B; and 11.0–12.0 min, 5% B with flow rate 0.5 mL/min. The column was operated at a constant temperature of 30 °C and the injection volume was 1 μL. All anthocyanins (cyanidin, cyanidin-3-galactoside, cyanidin-3-glucoside, cyanidin-3-arabinoside) were identified and quantified at λ = 520 nm wavelength.

### 3.13. High Performance Liquid Chromatography for the Determination of Sugars

The chromatographic analysis was carried out using a hydrophilic interaction liquid chromatography method (HILIC), using the Waters e2695 Alliance chromatographic system (Waters, Milford, MA, USA). Chromatographic separation was managed, chromatograms were recorded and data were processed with the software Empower^®^ v.3.0 (Waters, Milford, MA, USA). Chromatographic separations were carried out by using a Shodex SUGAR SZ5532 (150 × 6.0 mm, particle size 6 μm) column equipped with a Shodex SUGAR SZ-G (10 × 4.6 mm, particle size 6 μm) pre-column (Shodex Group, Tokyo, Japan). The volume of the injection was 10 µL. The flow rate was 1 mL/min, and gradient elution was used. The mobile phase consisted of water (solvent A) acetonitrile (solvent B). The following conditions of elution were applied: 0–5 min, 19% A; 5–20 min, 19–30% A; 20–22 min, 30% A; and 22–25 min, 30–19% A. The column was operated at a constant temperature of 60 °C. Detection was made with the evaporative light scattering detector (ELSD) Waters ACQUITY UPLC^®^ ELS Detector (Waters, Milford, MA, USA), with sprayer temperature—40 °C, nitrogen gas flow pressure—25 psi and evaporator temperature—60 °C. The identification of the chromatographic peaks was achieved by comparing the retention times and spectral characteristics of the eluting peaks with those of the reference compounds.

### 3.14. High Performance Liquid Chromatography Method for the Determination of Malic Acid

The chromatographic analysis was carried out using an ion-exclusion liquid chromatography method, using the Waters e2695 Alliance chromatographic system (Waters, Milford, MA, USA). Chromatographic separation was carried out by using a Shodex RSpak KC-118 (300 × 8.0 mm) column equipped with a Shodex RSpak KC-G (50 × 6.0 mm) pre-column (Shodex Group, Japan). The mobile phase consisted of 1 mM (*v/v*) perchloric acid in water, the volume of the injection was 10 µL and the flow rate was 1 mL/min. The column was operated at a constant temperature of 60 °C. Detection was made with the Waters 2489 UV/visible (UV/Vis) detector (Waters, Milford, MA, USA) at 210 nm. The identification of the chromatographic peaks was achieved by comparing the retention times and spectral characteristics of the eluting peaks with those of the reference compounds.

### 3.15. Determination of Antioxidant Activity Using HPLC-ABTS Post-Column Assays

The chromatographic separation of extracts was carried out by a Waters e2695 Alliance system (Waters, Milford, MA, USA) with a Waters 2998 photodiode array detector. The antiradical activity in extracts was evaluated using the HPLC-ABTS method as described by Raudonis et al. [66]. After detection, the eluate was directly mixed with radical cation ABTS˙+ reagent which was supplied to post-column by chromatograph “Beckman programmable solvent module 126” (Beckman, Fullerton, CA, USA) at a constant 0.5 mL/min flow rate. The reaction between analyte and reagent was carried out at the reaction coil, made out of polyether ether ketone (length 15 m, inner diameter 0.3 mm) under a temperature of 25 °C. The Waters 2487 detector was used to register the decrease of absorption after the reaction with ABTS˙+ (λ = 650 nm wavelength).

### 3.16. The Statistical Methods of Data Processing

All data were presented as an average of three years of study. Growing conditions in different years had an impact on the yield and fruit quality parameters. Meanwhile, differences between canopy positions remained the same and year and position interactions were not recorded for any of the tested parameters. Means and standard deviations were calculated with STATISTICA 10 StatSoft, Inc., Tulsa, OK, USA) and Excel (Microsoft, WA, USA) software. A one-way analysis of variance (ANOVA) along with the post-hoc Tukey’s HSD test was employed for statistical analysis. Differences were considered to be significant at *p* < 0.05.

## Figures and Tables

**Table 1:** Effect of canopy position on photosynthetic parameters, average 2019–2020.

Parameter	Canopy Position			
	Top	Inside	East	West
Photosynthetic rate, µmol CO_2_ m^−2^ s^−1^	18.35 ± 1.09 a *	10.79 ± 0.75 d	16.34 ± 1.00 b	12.92 ± 0.65 c
Stomatal conductance, mol H_2_O m^−2^ s^−1^	0.50 ± 0.05 a	0.30 ± 0.04 c	0.47 ± 0.05 ab	0.43 ± 0.05 ab
Intercellular CO_2_, µmol CO_2_ mol^−1^	300.2 ± 8.47 a	312.4 ± 9.74 a	306.9 ± 7.25 a	320.6 ± 3.45 a
Transpiration rate, mmol H_2_O m^−2^ s^−1^	5.23 ± 0.21 a	3.83 ± 0.38 c	5.02 ± 0.28 ab	4.81 ± 0.30 b
Chlorophyll index	40.5 ± 1.04 a	31.3 ± 3.10 c	34.2 ± 0.87 b	37.6 ± 2.21 ab
Flavonol index	1.89 ± 012 a	1.38 ± 0.16 c	1.77 ± 0.04 ab	1.72 ± 0.14 b
Nitrogen balance index	21.3 ± 1.36 b	23.1 ± 2.71 a	19.3 ± 0.62 c	22.1 ± 2.27 ab

* the different letters in the same line indicate significant differences between canopy positions (Tukey’s (HSD) multiple range test at the confidence level *p* = 0.05).

**Table 2:** Effect of canopy position on yield, fruit quality and maturity indices, average 2018–2020.

Parameter	Canopy Position			
	Top	Inside	East	West
Fruit yield, kg	8.1 ± 0.9 a *	6.3 ± 1.2 b	7.3 ± 1.0 ab	7.2 ± 0.8 ab
Fruit weight, g	190 ± 18.5 a	144 ± 12.4 c	158 ± 17.3 b	157 ± 18.2 b
Share of fruits with diameter >65 mm, %	96 ± 3.5 a	63 ± 8.2 c	81 ± 5.6 b	82 ± 5.9 b
Fruit blush, %	67 ± 5.2 a	22 ± 8.6 c	35 ± 7.2 cb	49 ± 8.3 b
Fruit firmness, kg cm^−2^	8.5 ± 1.01 a	8.2 ± 1.14 a	8.3 ± 1.21 a	8.6 ± 0.94 a
*I_AD_*	0.95 ± 0.08 a	0.83 ± 0.06 b	0.94 ± 0.08 a	0.99 ± 0.06 a
Starch index	7.9 ± 1.3 a	8.1 ± 1.1 a	8.2 ± 1.5 a	8.3 ± 1.2 a
Streif index	0.056 ± 0.011 a	0.055 ± 0.019 a	0.059 ± 0.023 a	0.057 ± 0.021 a
Respiration rate, mg CO_2_· Kg·h^−1^	2.2 ± 0.34 a	2.5 ± 0.22 a	2.3 ± 0.20 a	2.6 ± 0.32 a

* the different letters in the same line indicate significant differences between canopy positions (Tukey’s (HSD) multiple range test at the confidence level *p* = 0.05). *I_AD_*—*index* of absorbance difference.

**Table 3:** Effect of canopy position on fruit colour pattern according to CIE L*a*b* colour space coordinates, average 2018–2020.

Colour Coordinate	Canopy Position			
	Top	Inside	East	West
L*	33.6 ± 1.35 c *	55.7 ± 2.11 a	39.7 ± 1.56 b	38.8 ± 2.01 b
a*	31.8 ± 1.78 a	18.6 ± 2.31 b	32.1 ± 2.25 a	32.1 ± 2.32 a
b*	13.6 ± 1.25 c	29.3 ± 2.06 a	17.4 ± 1.51 b	16.5 ± 1.09 b
C*	35.4 ± 1.32 b	38.1 ± 2.01 a	36. 9 ± 1.07 a	36.1 ± 1.53 ab
h°	22.8 ± 1.51 c	63.6 ± 2.35 a	29.6 ± 2.22 b	27.5 ± 2.58 b

* the different letters in the same line indicate significant differences between canopy positions (Tukey’s (HSD) multiple range test at the confidence level *p* = 0.05).

**Table 4:** Effect of canopy position on fruit chemical content, average 2018–2020.

	Canopy Position			
	Top	Inside	East	West
Dry matter, %	15.2 ± 0.24 a *	14.4 ± 0.42 b	14.9 ± 0.36 ab	15.2 ± 0.27 a
Total sugar content, %	12.4 ± 0.09 a	11.7 ±0.29 b	12 ± 0.17 ab	12.1 ± 0.14 ab
Soluble solid content, %	13.5 ± 0.15 a	12.9 ± 0.32 a	13.3 ± 0.36 a	13.5 ± 0.20 a

* the different letters in the same line indicate significant differences between canopy positions (Turkey’s (HSD) multiple range test at the confidence level *p* = 0.05).

**Table 5:** Effect of fruit position in tree on apple sugar, sorbitol and malic acid composition, mg g^−1^ DW, average 2018–2020.

Compound	Canopy Position			
	Top	Inside	East	West
Fructose	447.2 ± 22.36 a *	505.6 ± 25.28 a	460.6 ± 23.03 a	493.9 ± 24.69 a
Glucose	82.9 ± 4.15 b	101.6 ± 5.08 a	87.5 ± 4.38 b	90.3 ± 4.52 ab
Sucrose	260.6 ± 13.03 a	229.2 ± 11.46 b	256.4 ± 12.82 a	251.5 ± 12.57 a
Sorbitol	23.2 ± 1.16 a	15.4 ± 0.77 c	18.9 ± 0.95 b	23.4 ± 1.17 a
Malic acid	40.2 ± 2.01 b	45.8 ± 2.29 a	42.7 ± 2.14 ab	40.1 ± 2.01 b

* the different letters in the same line indicate significant differences between canopy positions (Tukey’s (HSD) multiple range test at the confidence level *p* = 0.05).

**Table 6:** Effect of canopy position on fruit total phenolic, triterpene and anthocyanin content and radical scavenging activity, average 2018–2020.

Compound	Canopy Position			
	Top	Inside	East	West
Total anthocyanin content in apple peel, µg g^−1^ DW	177.35 ± 28.56 a *	19.33 ± 7.42 c	40.86 ± 14.91 bc	63.24 ± 12.67 b
Total phenolic content in whole apple, mg g^−1^ DW	2.41 ± 0.23 a	1.95 ± 0.12 b	2.18 ± 0.19 ab	2.09 ± 0.13 b
Total triterpene content content in apple peel, mg g^−1^ DW	12.65 ± 1.15 b	14.81 ± 1.22 a	13.52 ± 1.95 ab	13.01 ± 1.48 b
Radical scavenging activity (TE_ABTS_, μmol/g)	5.86 ± 1.10 a	4.20 ± 0.94 b	4.56 ± 0.88 ab	5.34 ± 1.07 a

* the different letters in the same line indicate significant differences between canopy positions (Tukey’s (HSD) multiple range test at the confidence level *p* = 0.05).

**Table 7:** Effect of canopy position on anthocyanin synthesis in apple fruits, mg g^−1^ DW, average 2018–2020.

Compound	Canopy Position			
	Top	Inside	East	West
Cyanidin-3-galactoside	150.84 ± 21.05 a *	15.34 ± 1.88 c	30.05 ± 8.68 bc	55.81 ± 3.66 b
Cyanidin-3-glucoside	0.17 ± 0.15 a	0.00 ± 0.00 a	0.00 ± 0.00 a	0.51 ± 0.57 a
Cyanidin-3-arabinoside	2.91 ± 1.76 a	0.00 ± 0.00 b	0.00 ± 0.00 b	0.26 ± 0.45 b
Cyanidin	3.16 ± 0.24 a	2.18 ± 0.14 b	2.19 ± 0.14 b	2.50 ± 0.37 b

* the different letters in the same line indicate significant differences between canopy positions (Tukey’s (HSD) multiple range test at the confidence level *p* = 0.05).

**Table 8:** Effect of canopy position on phenol composition, mg g^−1^ DW, average 2018–2020.

Compound		Canopy Position		
	Top	Inside	East	West
Hyperoside	0.18 ± 0.009 a *	0.04 ± 0.002 d	0.11 ± 0.006 b	0.10 ± 0.005 c
Isoquercitrin	0.04 ± 0.002 a	0.02 ± 0.001 c	0.03 ± 0.001 b	0.03 ± 0.002 b
Rutin	0.03 ± 0.001 a	0.01 ± 0.001 c	0.02 ± 0.001 b	0.02 ± 0.001 b
Reynoutrin	0.04 ± 0.002 b	0.01 ± 0.000 d	0.03 ± 0.001 c	0.05 ± 0.003 a
Avicularin	0.10 ± 0.005 a	0.04 ± 0.002 c	0.08 ± 0.004 b	0.09 ± 0.005 a
Quercitrin	0.11 ± 0.005 a	0.06 ± 0.003 c	0.09 ± 0.004 b	0.06 ± 0.003 c
Procyanidin B1	0.02 ± 0.001 b	0.03 ± 0.002 a	0.03 ± 0.001 b	0.02 ± 0.001 c
Procyanidin B2	0.48 ± 0.024 a	0.39 ± 0.019b c	0.41 ± 0.021 b	0.36 ± 0.018 c
Procyanidin C1	0.17 ± 0.008 a	0.15 ± 0.007 b	0.17 ± 0.008 a	0.16 ± 0.008 ab
(+)-Catechin	0.04 ± 0.002 a	0.04 ± 0.002 a	0.04 ± 0.002 a	0.03 ± 0.002 a
(−)-Epicatechin	0.29 ± 0.014 a	0.28 ± 0.014 a	0.30 ± 0.015 a	0.29 ± 0.015 a
Phlorizin	0.13 ± 0.006 a	0.11 ± 0.005 b	0.12 ± 0.006 ab	0.10 ± 0.005 b
Chlorogenic acid	0.79 ± 0.039 a	0.78 ± 0.039 a	0.77 ± 0.038 a	0.76 ± 0.038 a

* the different letters in the same line indicate significant differences between canopy positions (Tukey’s (HSD) multiple range test at the confidence level *p* = 0.05).

**Table 9:** Effect of canopy position on triterpene composition in apple peel, mg g^−1^ DW, average 2018–2020.

Compound	Fruit Position in Apple Tree Canopy			
	Top	Inside	East	West
Corosolic acid	1.25 ± 0.063 b *	1.44 ± 0.072 a	1.34 ± 0.067 ab	1.41 ± 0.071 ab
Betulinic acid	0.10 ± 0.005 c	0.14 ± 0.007 a	0.12 ± 0.006 b	0.09 ± 0.004 c
Oleonolic acid	1.89 ± 0.095 ab	2.13 ± 0.107 a	1.95 ± 0.098 ab	1.77 ± 0.088 b
Ursolic acid	8.65 ± 0.433 b	10.05 ± 0.502 a	8.90 ± 0.445 ab	8.65 ± 0.432 b

* the different letters in the same line indicate significant differences between canopy positions (Tukey’s (HSD) multiple range test at the confidence level *p* = 0.05).

## Data Availability

Not applicable.
